# Antibody Biomarkers Associated with Sterile Protection Induced by Controlled Human Malaria Infection under Chloroquine Prophylaxis

**DOI:** 10.1128/mSphereDirect.00027-19

**Published:** 2019-02-20

**Authors:** Joshua M. Obiero, Joseph J. Campo, Anja Scholzen, Arlo Randall, Else M. Bijker, Meta Roestenberg, Cornelus C. Hermsen, Andy Teng, Aarti Jain, D. Huw Davies, Robert W. Sauerwein, Philip L. Felgner

**Affiliations:** a Vaccine Research and Development Center, Department of Physiology and Biophysics, University of California, Irvine, Irvine, California, USA; b Radboud University Medical Center, Department of Medical Microbiology, Nijmegen, The Netherlands; c Antigen Discovery, Inc., Irvine, California, USA; University of Maryland School of Medicine; University of Copenhagen; University of Minnesota

**Keywords:** CHMI, antibody, malaria, preerythrocytic immunity, protein microarrays, sterile protection, vaccines

## Abstract

Immunization with sporozoites under chloroquine chemoprophylaxis (CPS) induces distinctly preerythrocytic and long-lasting sterile protection against homologous controlled human malaria infection (CHMI). To identify possible humoral immune correlates of protection, plasma samples were collected from 38 CPS-immunized Dutch volunteers for analysis using a whole Plasmodium falciparum proteome microarray with 7,455 full-length or segmented protein features displaying about 91% of the total P. falciparum proteome. We identified 548 reactive antigens representing 483 unique proteins. Using the breadth of antibody responses for each subject in a mixture-model algorithm, we observed a trimodal pattern, with distinct groups of 16 low responders, 19 medium responders, and 3 high responders. Fifteen out of 16 low responders, 12 of the 19 medium responders, and 3 out of 3 high responders were fully protected from a challenge infection. In the medium-responder group, we identified six novel antigens associated with protection (area under the curve [AUC] value of ≥0.75; *P* < 0.05) and six other antigens that were specifically increased in nonprotected volunteers (AUC value of ≤0.25; *P* < 0.05). When used in combination, the multiantigen classifier predicts CPS-induced protective efficacy with 83% sensitivity and 88% specificity. The antibody response patterns characterized in this study represent surrogate markers that may provide rational guidance for clinical vaccine development.

**IMPORTANCE** Infection by *Plasmodium* parasites has been a major cause of mortality and morbidity in humans for thousands of years. Despite the considerable reduction of deaths, according to the WHO, over 5 billion people are still at risk, with about 216 million worldwide cases occurring in 2016. More compelling, 15 countries in sub-Saharan Africa bore 80% of the worldwide malaria burden. Complete eradication has been challenging, and the development of an affordable and effective vaccine will go a long way in achieving elimination. However, identifying vaccine candidate targets has been difficult. In the present study, we use a highly effective immunization protocol that confers long-lasting sterile immunity in combination with a whole P. falciparum proteome microarray to identify antibody responses associated with protection. This study characterizes a novel antibody profile associated with sterile protective immunity and trimodal humoral responses that sheds light on the possible mechanism of CPS-induced immunity against P. falciparum parasites.

## Abstract (precis)

Infection by *Plasmodium* parasites has been a major cause of mortality and morbidity in humans for thousands of years. Despite the considerable reduction of deaths, according to the WHO, over 5 billion people are still at risk, with about 216 million worldwide cases occurring in 2016. More compelling, 15 countries in sub-Saharan Africa bore 80% of the worldwide malaria burden. Complete eradication has been challenging, and the development of an affordable and effective vaccine will go a long way in achieving elimination. However, identifying vaccine candidate targets has been difficult. In the present study, we use a highly effective immunization protocol that confers long-lasting sterile immunity in combination with a whole P. falciparum proteome microarray to identify antibody responses associated with protection. This study characterizes a novel antibody profile associated with sterile protective immunity and trimodal humoral responses that sheds light on the possible mechanism of CPS-induced immunity against P. falciparum parasites.

## INTRODUCTION

Developing a vaccine against malaria remains a formidable challenge, with over 4 decades of malaria vaccine clinical trials yielding only a single subunit vaccine, RTS,S/AS01 (Mosquirix), that has been approved (1). Although the RTS,S vaccine averts a substantial number of clinically symptomatic malaria infections in about 50% of children, this protection wanes several months after vaccination (2, 3). Difficulties with designing an effective vaccine for Plasmodium falciparum are compounded by a complex multistage life cycle, extreme genetic diversity, and highly evolved immune evasion mechanisms (4). The complexity of P. falciparum biology is further increased by its large 23-Mb genome encoding more than 5,300 different proteins (5, 6). Predicting which and how many of these proteins may induce protective immunity still remains an elusive challenge exacerbated by difficulties in culturing sporozoites and preerythrocytic stages of the parasite. Of the thousands of proteins expressed at any given time during the P. falciparum life cycle, only a few have been tested as vaccine candidates (7). Although both humoral and cellular responses have been associated with protection against malaria, there still remains the important question of which specific targets confer protection (8–11). Development of a vaccine benefits not only from immune correlates that predict protection but also from a clearer understanding of the immune mechanism of protection.

Advances in genomics, immunomics, and proteomics have benefited studies aimed at identifying immune correlates of protection and, more importantly, understanding the molecular immune mechanisms of protection (4, 5, 12). The use of controlled human malaria infection (CHMI) has enabled the development of very effective immunization protocols, such as immunization with whole viable sporozoites under chloroquine chemoprophylaxis (CPS), radiation-attenuated P. falciparum sporozoites (RAS), or genetically attenuated sporozoites (GAP) (13–15). Sterile homologous protection has been a hallmark of CPS immunization since the first immunization trial in 2009, in which 100% of the volunteers immunized with 45 mosquito bites acquired long-lasting immunity against malaria challenge infection (13, 16). In subsequent studies, we showed that this protection is dose dependent and predominantly preerythrocytic (17, 18).

Understanding the contribution of humoral immune responses in this immunization strategy is an important step toward elucidating the mechanism of protection and designing next-generation vaccine strategies. This has been made possible by the development of high-density whole-proteome microarrays that allow rapid interrogation of humoral responses against infectious diseases for vaccine antigen discovery and serodiagnostic antigen discovery (19–22). With over 5,300 protein-coding genes (5), possible interactions of P. falciparum with the human immune system are complex, and it is challenging to identify parasite antigens that induce protective immune responses. In a previous study, we probed a partial P. falciparum proteome microarray containing 1,000 protein features and reported that CPS immunization induces a distinct antibody (Ab) profile compared to one induced by naturally acquired infection (22). For the present study, we produced a more complete proteome array containing 7,455 features representing 4,805 P. falciparum genes covering 91% of the P. falciparum proteome. Here, we tested specimens from 3 consecutive CPS immunization trials with subsequent homologous challenge, involving a total of 30 fully protected and 8 nonprotected individuals. Differences in Ab profiles between protected and nonprotected volunteers identified antigens associated with CPS-mediated protective immunity and others associated with susceptibility to infection. The antigens associated with protective immunity can be considered potential candidates for a multiantigen preerythrocytic vaccine to induce sterile protective immunity.

## RESULTS

### CPS immunization induces broad and variable humoral responses.

**FIG 1:**
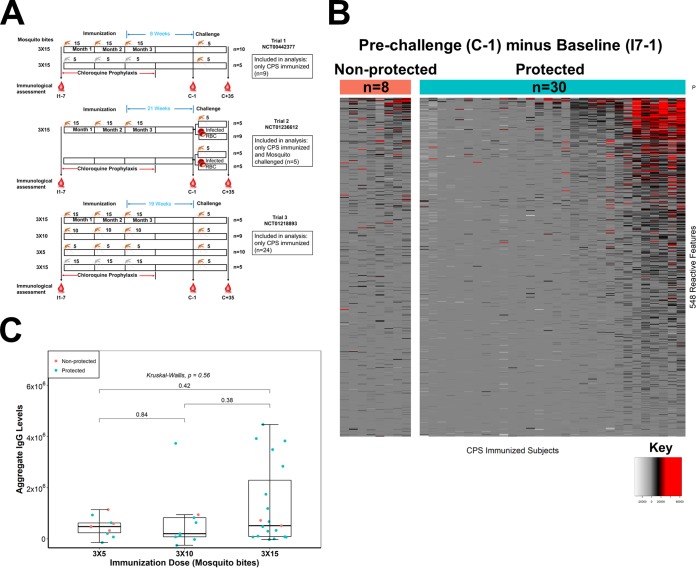
CPS immunization induces broad and variable humoral responses against P. falciparum antigens. (A) Plasma samples from 38 CPS-immunized volunteers from 3 clinical trials (*n* = 9 from study 1, *n* = 5 from study 2, and *n* = 24 from study 3) were probed on whole P. falciparum proteome microarrays. Thirty CPS-immunized volunteers were protected and eight were not protected from malaria challenge. RBC, red blood cells. (B) Plasma samples collected preimmunization (I1-7) and postimmunization/prechallenge (C-1) were probed, and signals were quantified. The heat map represents the signal intensity at time point C-1, corrected for background reactivity by subtracting the signal intensity at I1-7, against 548 immunogenic P. falciparum features (or 483 unique proteins) printed on proteome microarrays. For an antigen to be considered immunogenic, (i) its levels had to have increased 100% at C-1 compared to I1-7 and (ii) its seroprevalence had to be >15%. The specimens are ordered in clinical groups, nonprotected (*n* = 8) and protected (*n* = 30). Samples are arranged with increasing reactivity from left to right, while the antigens are arranged with decreasing reactivity from top to bottom. A color gradient is used to display the signal intensity detected for each antigen, as shown by the key. (C) Comparison of aggregated C-1 reactivity of the 548 immunogenic antigens among the 3 different immunization dose groups. During the 3 rounds of immunization, 10, 9, and 19 volunteers received 5, 10, and 15 mosquito bites, respectively. Dots show individual data points, center lines show the medians, box limits indicate the 25th and 75th percentiles, and whiskers extend 1.5 times the interquartile range (IQR). Aggregate antibody levels were compared using the Kruskal-Wallis rank sum test. To detect only malaria-specific responses, the I1-7 responses were subtracted from the C-1 responses.

Plasma specimens from 38 volunteers enrolled in 3 clinical CPS immunization trials were collected at 3 time points: (i) preimmunization (7 days before CPS immunization [I1-7]), (ii) postimmunization (1 day before CHMI challenge [C-1]), and (iii) postchallenge (35 days postchallenge [C+35]) (Fig. 1A). The heat map in Fig. 1B shows 548 immunogenic P. falciparum antigens that met the following two criteria: (i) seropositivity in more than 15% of all the CPS-immunized volunteers (*n* = 38) and (ii) antibody levels doubled at C-1 compared to I1-7. Signal intensities measured at I1-7 were subtracted from signal intensities at C-1 to control for background reactivity, and these data were then used to create the heat map. The antibody reactivity profiles of the 38 CPS-immunized individuals revealed great variability, with some volunteers showing intense reactivity (high antibody levels) against a large number of antigens and others reacting against a small number of antigens (Fig. 1B). Fourteen antigens were recognized by at least 50% of the volunteers, with the top five most commonly recognized antigens being CSP (89% of volunteers), LSA1 (82%), LISP2 (71%), EXP1 (66%), and MSP2 (63%) (see Table S1 in the supplemental material).

10.1128/mSphereDirect.00027-19.1 TABLE S1 Plasmodium falciparum proteins printed on the microarrays. Shown is a list of all proteins printed on the microarray. Gene identifications, gene product descriptions, protein lengths, and the presence of transmembrane domains and signal peptides according to PlasmoDB are shown. Additionally, the numbers of nonprotected and protected subjects reactive and the seroprevalence of a specific feature are indicated. Proteins with a seropositive response in at least 25% of the subjects in a group and with mass spectrometry evidence of protein expression from PlasmoDB are shown. Download Table S1, XLSX file, 0.7 MB. Copyright © 2019 Obiero et al. 2019 Obiero et al. This content is distributed under the terms of the Creative Commons Attribution 4.0 International license.

For this analysis, specimens from 3 studies were combined to create 3 dose groups. The first group of 10 volunteers received a total of 15 infectious mosquito bites, the second group of 9 volunteers received 30 mosquito bites, and the third group of 19 volunteers received 45 mosquito bites over a period of 3 months (Fig. 1A). To study the effect of immunization dose on antibody response, we summed up each individual’s response against the 548 immunogenic antigens (Fig. 1C). There was no significant difference in the aggregate P. falciparum antigen-specific IgG levels between the 3 mosquito bite dose groups (*P* = 0.56 by the Kruskal-Wallis test). Since the dose of immunization does not account for the observed antibody reactivity differences in the subsequent analyses, we used two metrics, “breadth” and “magnitude,” to further assess the association between the antibody responses and protection.

### CPS immunization induces trimodal IgG responses.

**FIG 2:**
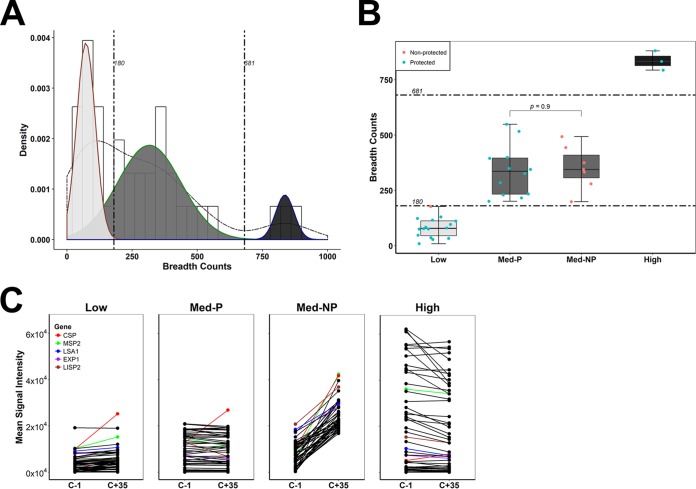
CPS immunization induces trimodal IgG responses in previously malaria*-*naive adults. (A) Three-component mixture model for breadth counts observed in CPS-immunized individuals (*n* = 38). The solid curved lines show the fitted breadth count distributions in the mixture model. The dashed curve line shows the mixture-model fit for components 1 (orange), 2 (blue), and 3 (green). The two dashed straight lines show the estimated cutoff points at 180 and 681 antigens, which distinguish the different responder groups (low, medium, and high responders). The histogram represents the frequency of the breadth counts in the different bins. Breadth counts are ranked from the lowest to the highest (range, 9 to 881). (B) Box plots of low-, medium-, and high-responder groups showing protected and nonprotected volunteers. Comparison of breadth counts within the medium-responder group was performed by a Wilcoxon rank sum test. Gray dotted lines show the cutoff points between responder groups. Dots show individual data points, center lines show the medians, box limits indicate the 25th and 75th percentiles, and whiskers extend to show the minimum and maximum breadth counts. (C) Humoral reactivity was compared prechallenge (C-1) and post-CHMI challenge (C+35). Mean antibody signal intensities measured against the top 50 most reactive antigens after CHMI challenge are shown, comparing low, medium, and high responders. The medium-responder group is further stratified into protected (“Med-P”) and nonprotected (“Med-NP”) volunteers. The mean reactivities of the top 5 most seroprevalent antigens, CSP, LSA1, LISP2, EXP1, and MSP2, are shown. Black dotted lines indicate the other boosted antigens after challenge (see Table S2 in the supplemental material).

In the present study, a two-component mixture model was used to determine the breadth count for each individual specimen (see Materials and Methods and Fig. S1). There was considerable interindividual variation in the breadths of the induced antibody specificities, with some individuals reacting to as few as 9 antigens, while the strongest responder reacted to 881 antigens. Some of the subjects had low responses to a small number of antigens, while others had high responses to many antigens. We constructed and applied a three-component mixture model on the breadth counts of all the subjects, which clustered the different distributions of antibody breadth profiles into three distinct groups (Fig. 2A). Using this model, two cutoff points (180 and 681 antigens) were identified, which separated the three groups of responders. One group responded to a limited number of antigens (breadth range, 9 to 177) (low responders). The second group reacted to a moderate number of antigens (breadth range, 199 to 549) (medium responders), while the third group was highly reactive against a large number of antigens (breadth range, 794 to 881) (high responders) (Fig. 2B). Fifteen of the 16 low responders, 12 of the 19 medium responders, and all the high responders were protected from malaria challenge (Fig. 2B). Antibody reactivity at C+35 in protected individuals, irrespective of their responder status, was stable or declined compared to C-1 levels, except for reactivity against CSP, which increased in all groups (Fig. 2C). In contrast, nonprotected individuals showed a markedly increased response upon challenge, consistent with data from other studies (20, 23); the appearance of new antigens from a productive infection leads to a robust antibody response against blood-stage antigens, whereas sterile protection prevents the presentation of new blood-stage antigens to the immune system, and responses do not change after challenge. Low responder referred to all subjects who recognized fewer than 180 proteins. However, it is important to note that low responders also had lower average signal intensities seen at C-1 and C+35 (Fig. 2C) against the top 50 most reactive antigens after challenge (Table S2).

10.1128/mSphereDirect.00027-19.4 FIG S1 Raw microarray antibody reactivity. (A) Bar plots showing representative samples at the C-1 time point. Humoral reactivity against 7,455 P. falciparum antigens on the microarray is sorted from most reactive to least reactive. The black dotted line shows the cutoff point obtained using a mixture-model analysis. (B) Each subject’s humoral response data from all time points were analyzed using a mixture model. This allowed for identification of positive (blue curved line) and negative (yellow curved line) signal intensity distributions in an overall population of signals. The mean of the negative signal distribution plus 3 standard deviations is used as a cutoff point (shown here as a dotted black line). This individualized cutoff point is applied to data from each time point (C-1 shown in panel A) to identify reactive antigens in each of the samples. For each individual sample, the number of seropositive antigens was counted to determine the breadth count. For better visualization, the horizontal axis scale was transformed using the function sqrt_trans() in the R package “scales.” Download FIG S1, TIF file, 0.5 MB. Copyright © 2019 Obiero et al. 2019 Obiero et al. This content is distributed under the terms of the Creative Commons Attribution 4.0 International license.

10.1128/mSphereDirect.00027-19.2 TABLE S2 Antigens boosted after challenge in nonprotected volunteers. Download Table S2, TIF file, 1.9 MB. Copyright © 2019 Obiero et al. 2019 Obiero et al. This content is distributed under the terms of the Creative Commons Attribution 4.0 International license.

### Stage of expression of proteins recognized by CPS-immunized subjects.

**FIG 3:**
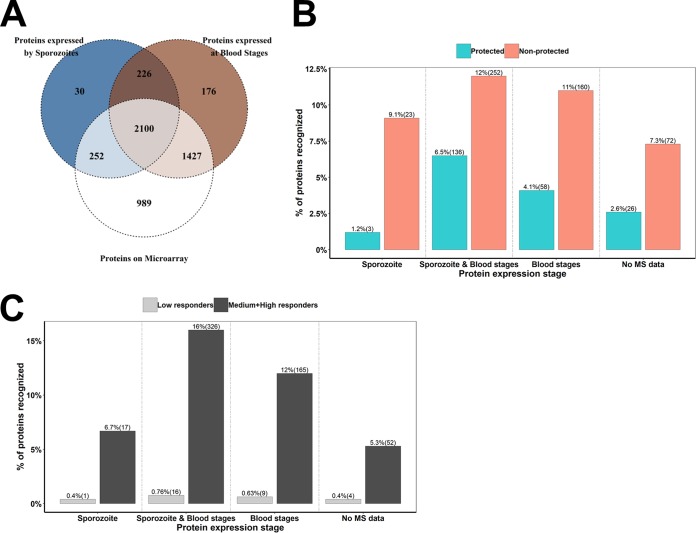
Stage of expression of proteins printed on the microarray. Publicly available tandem mass spectrometry (MS/MS) data on protein expression were obtained from PlasmoDB. (A) Venn diagram showing stage-specific profiles of the proportions of proteins expressed by sporozoite and blood stages with MS/MS evidence of expression and printed on the microarray. (B and C) Bar plots showing numbers of P. falciparum proteins uniquely or commonly expressed at sporozoite and/or blood stages and recognized by at least 25% of nonprotected or protected volunteers (B) or low, medium, and high responders (C). The names and gene annotations of these proteins are indicated in Table S1 in the supplemental material.

In order to characterize the distribution of the proteins recognized by CPS-immunized subjects at different stages of the P. falciparum life cycle, we downloaded protein annotations from PlasmoDB with tandem mass spectrometry (MS/MS) evidence for expression (see Materials and Methods). Of the 5,305 protein-coding genes annotated in PlasmoDB (6), MS/MS evidence of expression is available for 4,216 proteins. We aligned the Ab reactivities with this MS evidence of expression and tabulated (Table S1) and plotted (Fig. 3) the results. Of the 4,768 unique proteins printed on the microarray, 252 and 1,427 are exclusively expressed by sporozoites and blood-stage parasites, respectively. A total of 2,100 antigens are commonly expressed at both stages, while 989 proteins recognized by CPS-immunized subjects lack MS evidence of expression (Fig. 3A).

Overall, nonprotected subjects recognized more antigens than protected subjects at all stages tested (Fig. 3B and Table S1). Nonprotected subjects recognized 9% (23) and 11% (160) of the proteins expressed uniquely at sporozoite and blood stages, respectively. They also recognized 12% (252) of the proteins expressed at both stages (Fig. 3B). On the other hand, protected subjects recognized 1% (3) of the proteins uniquely expressed by sporozoites, 4% (58) of the proteins exclusively expressed at blood stages, and 6.5% (136) of the proteins expressed at both stages and printed on the array (Fig. 3B). As expected, medium and high responders reacted to more antigens than did low responders. Most of the antigens recognized by medium and high responders are commonly expressed at sporozoite and blood stages (Fig. 3C). Interestingly, CSP is the only sporozoite protein recognized by low responders. Furthermore, they respond to 0.6% (9) of the proteins expressed at the blood stage and 0.8% (16) of the proteins expressed at both stages (Fig. 3C). Medium and high responders recognized 8% (17), 16% (326), and 12% (165) of the proteins expressed at the sporozoite stage, the blood stage, and both stages, respectively. The gene annotations and protein names are shown in Table S1.

### Novel antibodies associated with CPS-induced sterile protection.

Our data show trimodal humoral responses with low, medium, and high responders. We hypothesized that protected low responders were protected from CHMI by cellular responses against sporozoite and early-liver-stage antigens, preventing exposure to late-liver-stage and blood-stage antigens. This low parasite load therefore results in the low breadth count. On the contrary, medium/high responders do not clear early-liver-stage parasites, the parasites progress through later stages, and the subjects acquire higher breadth counts. Based on this hypothesis and the fact that there was only one nonprotected subject in the low- and high-responder groups, we analyzed antibody responses associated with protection in the medium-responder group only.

**FIG 4:**
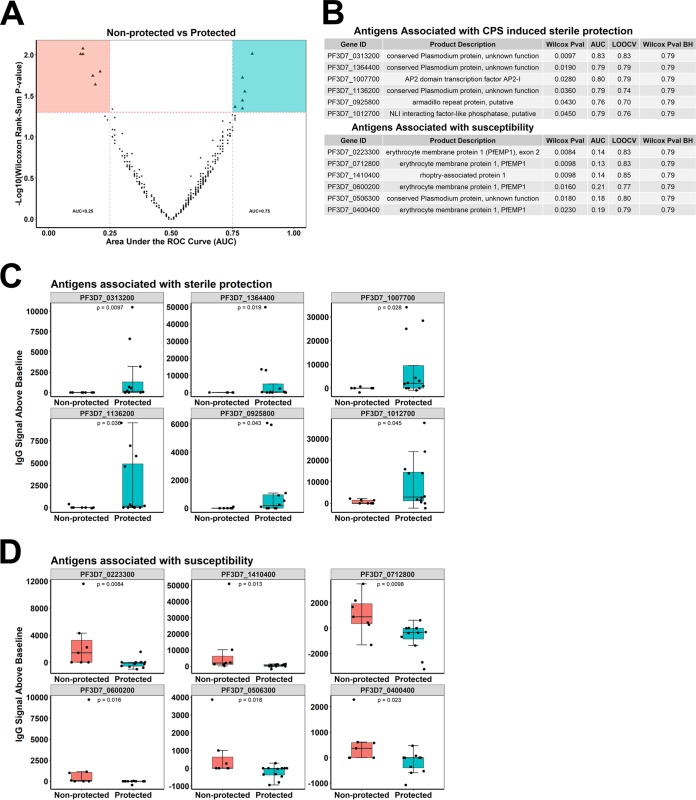
Novel antigens associated with CPS immunization*-*induced sterile protection. (A) Differential signal intensities of 229 antigens at C-1 in plasma samples of protected (*n* = 7) and nonprotected (*n* = 12) medium responders. The red dotted line indicates the threshold *P* value of <0.05; dots in the green area and dots in the orange area represent antigens significantly more reactive in protected and nonprotected subjects, respectively. (B) Gene identifications of the antigens associated with protection and susceptibility in medium responders. Wilcoxon rank sum test *P* values (Wilcox Pval), Wilcoxon rank sum test *P* values with Benjamini-Hochberg correction for multiple comparisons (Wilcox Pval BH), area under the receiver operating characteristic curve (AUC) values, and leave-one-out cross-validation (LOOCV) AUC values for protected and nonprotected volunteers are shown. (C and D) Specific antigens associated with protection (C) or susceptibility (D). Boxes represent individual antigens in nonprotected (orange) (*n* = 7) and protected (green) (*n* = 12) subjects. Dots show individual data points, center lines show the medians, box limits indicate the 25th and 75th percentiles, and whiskers extend 1.5 times the IQR. Comparisons were tested by a Wilcoxon rank sum test.

Antibody targets associated with sterile protective immunity were identified by comparing 7 nonprotected and 12 protected medium responders (Fig. 2B). All 548 immunogenic antigens were individually tested in univariate analysis by (i) performing a Wilcoxon rank sum test (Fig. 4A) (with multiple-testing correction) and (ii) constructing receiver operating characteristic (ROC) curves and calculating the area under the ROC curve (AUC) values for each antigen (Fig. 4A). Using an AUC value cutoff of ≥0.75 and a Wilcoxon *P* value of <0.05, we identified antibody responses against six novel P. falciparum-specific targets associated with sterile protection (Fig. 4A and B); we also identified six antigens that had significantly increased antibody responses in nonprotected individuals, with an AUC value of ≤0.25 and a Wilcoxon *P* value of <0.05 (Fig. 4A and B). CPS-immunized subjects are exposed to parasitemia during the first immunization, and in some subjects, there is recurrent exposure to parasitemia in subsequent immunizations. Even though both protected and nonprotected subjects undergo similar infections during immunization, protected individuals do not recognize four of the antigens associated with susceptibility, which is indicative of protection status. In comparison, nonprotected subjects show higher-magnitude reactivity to all six antigen “susceptibility markers” (Fig. 4D). In order to check for generalizability of the data from ROC analysis, we also performed leave-one-out cross-validation (LOOCV) analysis on the two sets of antigens (Fig. 4B). The magnitude of signal intensities for each of the six antigens associated with protection was significantly higher in protected than in nonprotected volunteers (Fig. 4C). Inversely, the six antigens associated with susceptibility to malaria showed a higher magnitude in nonprotected than in protected individuals (Fig. 4D). We also repeated the above-described univariate analysis on the immunogenic antigens after excluding all antigens with MS evidence of expression at blood stages. Of the 483 unique immunogenic proteins on the array, 149 of these were exclusively expressed at blood stages. Therefore, the analysis was performed on 334 proteins represented by 391 features on the array. Five of the six antigens (PF3D7_0313200, PF3D7_1007700, PF3D7_1012700, PF3D7_1364400, and PF3D7_0925800) associated with protection in our previous analysis were also identified by this analysis (data not shown). Even though the well-studied antigens CSP, LSA1, LISP2, EXP1, and MSP2 were highly seroprevalent and in some studies used as vaccine targets (2, 3, 24–26), they were not associated with protection or susceptibility either individually or in combination (Fig. S2).

10.1128/mSphereDirect.00027-19.5 FIG S2 Antibodies to top immunogenic antigens are not associated with protection. (A) Box plots showing comparisons of these antigens between protected (green) (*n* = 12) and nonprotected (red) (*n* = 7) subjects. Dots show individual data points, center lines show the medians, box limits indicate the 25th and 75th percentiles, and whiskers extend 1.5 times the IQR. (B) Receiver operating characteristic (ROC) curves were used to test individual antigens for association with protection. The performances of the top five most immunogenic antigens with an area under the receiving operating characteristic curve (AUC) are shown. LOOCV analysis was also performed for these antigens. An AUC value of 0.5 indicates that there is no difference between the groups. Download FIG S2, TIF file, 0.5 MB. Copyright © 2019 Obiero et al. 2019 Obiero et al. This content is distributed under the terms of the Creative Commons Attribution 4.0 International license.

### The breadth of antibodies classifies protected and nonprotected volunteers.

**FIG 5:**
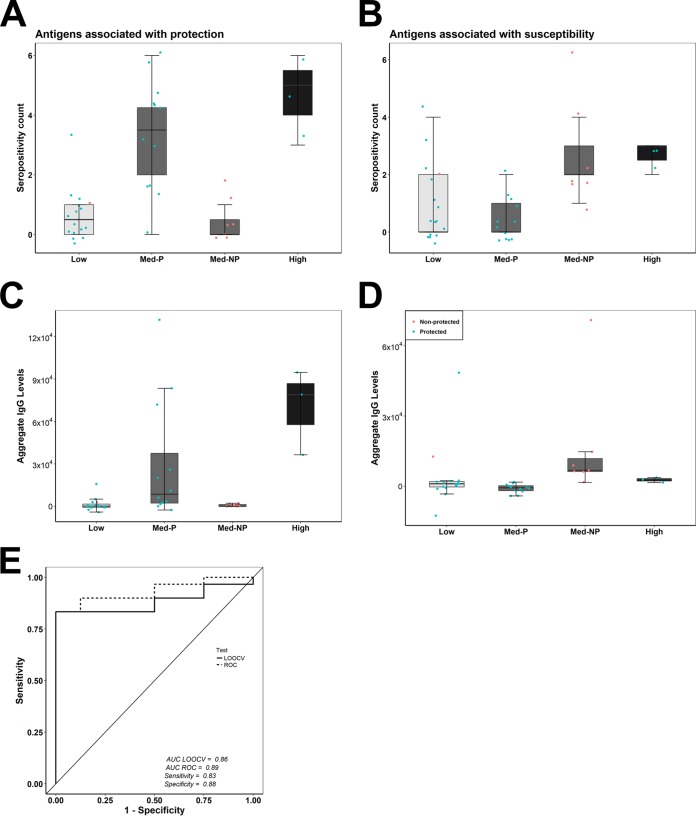
Breadth of antigen response as a correlate of protection. (A and B) Breadth counts of protective (A) and nonprotective (B) antigens in protected (*n* = 30) and nonprotected volunteers (*n* = 8). Dots show individual data points, center lines show the medians, box limits indicate the 25th and 75th percentiles, and whiskers extend 1.5 times the IQR. Antibody intensities were compared using the Wilcoxon rank sum test. (C) The difference between protective and susceptible seropositivity counts was used to construct an ROC curve. LOOCV analysis was also performed. An AUC value of 0.5 indicates that there is no difference between the groups.

We compared protective and susceptible seropositivity counts (Fig. 5A and B and Table S3). As expected, protected medium responders had higher protective seropositivity counts than nonprotected medium responders. The reverse was true for susceptible seropositivity counts. We further compared the aggregate IgG responses associated with protection and susceptibility (Fig. 5C and D). The aggregate IgG responses associated with protection (Fig. 5C) and those associated with susceptibility (Fig. 5D) were higher in protected and nonprotected medium responders, respectively. High responders (all protected) had the highest protective seropositivity counts and similarly the highest aggregate IgG responses. Although high responders had susceptible seropositivity counts similar to those of nonprotected medium responders, the aggregate IgG levels were much lower. Low responders had low responses on both metrics. To test whether the difference between protective seropositivity counts and susceptible seropositivity counts could discriminate protected from nonprotected CPS-immunized subjects, we performed both ROC curve and LOOCV analyses. We constructed an ROC curve after subtracting susceptible seropositivity counts from protective seropositivity counts for each subject, which predicted protected subjects with 83% sensitivity and 88% specificity (AUC value of 0.89) and an LOOCV AUC value of 0.86 (Fig. 5E). The combined results suggest that these two distinct classes of antigens can be used together in a serodiagnostic test to predict outcomes after CPS immunization.

10.1128/mSphereDirect.00027-19.3 TABLE S3 Breadth counts and protective and susceptible seropositivity counts. Download Table S3, TIF file, 0.9 MB. Copyright © 2019 Obiero et al. 2019 Obiero et al. This content is distributed under the terms of the Creative Commons Attribution 4.0 International license.

## DISCUSSION

Using a comprehensive P. falciparum protein microarray covering approximately 91% of the P. falciparum proteome, we show that the antibody specificities of CPS-immunized volunteers react to a broad repertoire of antigens, with substantial interindividual variability in both breadth and magnitude. We identified 548 antigens (fragments) reactive in more than 15% of the CPS-immunized individuals, which represent 483 unique P. falciparum proteins. The breadths of the antibody responses among protected individuals varied from recognition of as few as nine to several hundred antigens. The breadths of the responses showed a trimodal distribution, and immunized individuals could be classified into high, medium, and low responders. Our data show that protection is not directly associated with breadth of response, as 15 out of 16 low responders were protected. From the medium-responder group, six antigens stand out, which were significantly more reactive in protected individuals, while levels of another 6 “susceptibility antigens” were elevated in nonprotected individuals.

To better quantify antibody breadth and define the positivity of a signal, we developed a new computational approach that takes the variable baseline reactivity between individuals into consideration. The approach uses a mixture model where all antigens’ signal intensities were used to model two distributions (low and high). The mean and standard deviation (SD) of the lower distribution were used to set the threshold for classifying seropositive antibodies for that sample. In our previous studies, seropositive responses were defined by creating a cutoff point using the mean reactivity against *in vitro* transcription and translation (IVTT) controls from all volunteers in the study (global threshold). This IVTT control signal intensity is proportional to the amount of anti-Escherichia coli Ab in the sample, which varies depending on the individual. Importantly, our approach uses antimalaria reactivity for defining the threshold rather than the anti-E. coli reactivity that was used previously (19–21). While care is taken to block all reactivity against E. coli antigens, depending on the subjects’ disease history, this varies between subjects. Therefore, the use of anti-P. falciparum responses rather than reactivity against E. coli as the basis for specifying seropositivity makes the analysis more specific for P. falciparum. The new method treats each volunteer individually, thereby limiting “missed reactive antigens” in low-responding volunteers, which would occur when a global median cutoff is used.

Here, we show that CPS immunization is associated with an interesting dichotomy between the breadth of seropositive antigens and sterile protection; 42% of the volunteers develop low breadth counts, while 58% have intermediate to high breadth counts. While this unpredicted pattern does not correlate with protection, it shows how variable humoral responses against P. falciparum can be. While our study provides insights into immune-mediated protection from malaria, it also shows that antibody responses are associated with protection in about 50% of the subjects. Immune response variability in CHMI has also been shown by Burel et al., who reported high expression levels of a set of microRNAs that correlated with high cellular and antibody responses seen in CHMI volunteers (27). These studies highlight the need for a better understanding of immune responses against malaria. It remains to be seen whether the observed variable patterns in CPS-immunized subjects are driven by genetics, age, or other factors.

Interestingly, 15 of the 16 volunteers classified as low responders were protected from CHMI in spite of the low breadth counts. In contrast, 7 out of the 8 nonprotected individuals had higher breadth counts than the protected low responders. It is noteworthy that the 1 nonprotected individual who was classified as a low responder actually had the highest breadth count of that group (177), close to the breadth count cutoff of 180, which would put this individual into the medium-responder category. There was no significant difference in the breadth counts between protected and nonprotected medium responders; however, their overall responses to challenge were distinctly different. A likely explanation is that cellular immune responses against early-liver-stage development protect these volunteers in the humoral low-responder group, resulting in reduced exposure to later-stage P. falciparum antigens and, hence, limited antibody reactivity to a small repertoire of antigens. Indeed, production of gamma interferon (IFN-γ), tumor necrosis factor (TNF), and interleukin-2 (IL-2) by CD4^+^ T cells has been associated with preerythrocytic protection following CPS immunization and natural infection (13, 28, 29). However, it has also been reported that antibodies from CPS-immunized and protected human volunteers inhibit sporozoite traversal through hepatocytes and reduce liver-stage infection in the human liver chimeric mouse model. Protected medium and high responders may be protected by a broader repertoire of antibodies against late-liver-stage parasite antigens. Indeed, low responders show significantly lower antibody responses against the mid/late-liver-stage antigens LSA1 and LISP2 than medium/high responders. There was no difference in reactivities against the sporozoite/early-liver-stage antigen CSP and a blood-stage antigen, EXP1. EXP1 is also expressed at the liver stage, and it contains 15-amino-acid region of homology to the NANP repeat sequence on CSP, an immunodominant epitope shown to be responsible for cross-reactivity (30, 31). Therefore, this may explain the similar reactivities in low and medium/high responders against both CSP and EXP1. MSP2, a blood-stage antigen, is not recognized in low responders but is well recognized in the medium/high responders (32). Even though anti-CSP antibodies are not associated with protection in the present study, immune responses against CSP or other intrinsic sporozoite antigens may limit infection of liver cells, leading to low-level recognition of other liver-stage/blood-stage antigens and the low-responder phenotype.

Despite the fact that chloroquine works by preventing parasites from developing in red blood cells (33), CPS-immunized subjects still recognize more blood-stage-expressed proteins than those coexpressed at the sporozoite stage. This could be explained by the fact that during immunization, there is exposure to the first wave of mature and fully developed blood-stage parasites emerging from infected liver cells (13, 18, 34). In addition, compared to blood stages, sporozoites are seen for only a few hours while in the bloodstream before reaching the liver, where they grow and divide. The antigenic load favors the blood stages, as each infected liver cell releases tens of thousands of merozoites into the bloodstream (35, 36). Due to the technical difficulties involved in culturing preerythrocytic stages of P. falciparum, there should be more protein expression studies conducted on blood stages of P. falciparum, which can be cultured with relative ease.

Whether or not responses against these proteins exclusively expressed at blood stages are important in CPS-induced protection is not known. The exclusion of proteins exclusively expressed in blood stages does not drastically change the results of our analysis. Only one of the proteins identified is expressed at blood stages according to MS data (see Table S1 in the supplemental material). In order to capture antigens associated with both protection and susceptibility, we performed univariate analysis on all immunogenic proteins. It is important to note that the MS data available are not fully comprehensive, and there are hundreds of proteins with no expression data. The addition of more expression data will greatly benefit the comparison of stages of expression and immunogenicity.

Naturally acquired immunity (NAI) is associated with increased breadth and intensity of the humoral response against dozens of blood-stage parasite antigens and increased protection from clinical malaria, but NAI does not induce sterile immunity (22, 37). In contrast, most of the sterilely protected CPS-immunized volunteers are able to control liver-stage parasites after 2 rounds of immunization, and as a consequence, they do not have measurable blood-stage parasites after the third immunization (18, 34). In the present study, we show that in volunteers with sterile protection from challenge, there is no boosting of specific antibodies except for CSP, which indicates exposure of these volunteers only to sporozoites but not to blood-stage parasites during challenge. This finding is consistent with the induced preerythrocytic immunity that prevents parasitemia (18, 34). Conversely, nonprotected volunteers have increased reactivity against hundreds of antigens after challenge, which indicates exposure to both sporozoites and blood stages. These results corroborate data from our previous studies showing that protected subjects boost antibody responses only to CSP and that nonprotected volunteers undergo recurrent parasitemia during immunizations (17, 34). This also explains why nonprotected volunteers have significantly higher antibody responses against antigens associated with susceptibility, 5 out of 6 of which are well-characterized blood-stage antigens. The number of mosquito bites during CPS immunization is an important denominator for blood-stage exposure during immunization in both protected and nonprotected volunteers (17, 34). Protection, at least for the medium-responder group, seems to be associated with antibody responses against a panel of six antigens. Even though low responders had a minimal-magnitude response to a few antigens, 94% of them were protected from malaria challenge. Interestingly, low responders boost reactivity against CSP only after challenge, suggesting that the immune system is able to respond to and clear the sporozoites quickly after infection. Previously, Bijker et al. showed that protection after CPS immunization was associated with higher proportions of CD4 and CD8 cytotoxicity markers (17). We did not see an association of protection in low responders with responses against the set of antigens identified in the medium-responder group. High responders were highly reactive against hundreds of antigens, and the synergistic effect of responses against these antigens could play a role in the protection seen.

Of note, several studies have associated both the breadth and magnitude of antibody responses with protection against malaria (37, 38). In the present study, responses against MAEBL, TRAP, and SEA1 were not associated with protection after CPS immunization, in contrast to a previous study conducted on samples from CPS-immunized individuals (38). The most intensely reactive antigens following CPS immunization, CSP, LSA1, LISP2, EXP1, and MSP2, are well-established vaccine candidates. Previously, our group has shown that CSP, LSA1, and MSP2 are highly reactive after CPS immunization (22). Furthermore, both LSA1 and LISP2 have been shown to be highly reactive in a PfSPZ-CVac vaccine trial (39). Our study shows that antibodies against these highly immunogenic antigens were not associated with CPS-mediated protection in combination or individually. This indicates that, at least in the case of CPS immunization, antibody responses against these immunodominant antigens do not necessarily equate with protection, but they may be markers of exposure. This is interesting in light of diverse results in both human and animal trials with CSP, the major vaccine candidate in clinical development (2, 3, 40, 41).

Importantly, protection in this study is associated with responses against six novel antigens, three of which are conserved proteins with unknown function, an armadillo repeat protein (PF3D7_0925800), NLI-interacting factor-like phosphatase (PF3D7_1012700), and a transcription factor (PF3D7_1007700). Of the conserved proteins, PF3D7_0313200 is predicted to be a DNA binding protein. The are no MS/MS data available for this protein, although the transcript has been detected in both sporozoites and gametocytes by transcriptome sequencing (RNA-Seq) studies. It is thought to be located on the cell surface, cytoplasm, or apicoplast (42). PF3D7_1136200 has a signal peptide and is predicted to be an anchored component of the plasma membrane. There is evidence from RNA-Seq studies showing that it is expressed by sporozoites, late trophozoites, and gametocytes. However, MS/MS studies show that the protein is present only in merozoites and schizonts (6, 42–45). PF3D7_1364400 has two transmembrane domains and an extracellular region; MS/MS and proteome studies have shown that it is expressed by both sporozoites and blood stages (6, 46, 47). PF3D7_0925800 is predicted to be located in the cytoplasm and is involved in RNA metabolic processes. Its expression has been shown in sporozoites, trophozoites, and gametocytes (6, 46, 48, 49).

PF3D7_1012700, a member of the NIF family, is located in the nucleus and has been shown to be important in drug responses (6, 50–52). This protein has been found in sporozoite, schizont, trophozoite, and gametocyte life stages (43, 47, 53). The 26-member AP2 gene family has been shown to have a variety of roles in gene expression (54–56). Specifically, PF3D7_1007700, a member of the ApiAP2 family of DNA binding proteins, is associated with the regulation of genes involved in invasion (56–58). Both MS/MS and RNA-Seq data show that this protein is expressed in sporozoites and blood stages of the parasite (6, 42, 49, 53, 59–61). Interestingly, another member of this family, PF3D7_0420300, was preferentially more reactive in protected volunteers although with borderline significance (*P* = 0.059). Moreover, the antibody response against PF3D7_1139300, another ApiAP2 gene, was associated with protection in a study where individuals were immunized with irradiated sporozoites via bites from infected mosquitoes (23).

Our study has identified 6 antigens associated with susceptibility and 6 antigens associated with protection. When used together, the combined set predicts protection among all the CPS-immunized volunteers with 83% sensitivity and 88% specificity.

The 6 previously unrecognized antigens associated with sterile protection in this study are potential vaccine antigen targets.

## MATERIALS AND METHODS

### Study population.

Studies from our group have shown that immunization of healthy human volunteers via bites of P. falciparum-infected mosquitos under chloroquine prophylaxis (CPS immunization) induces sterile preerythrocytic protection against controlled human malaria infection (CHMI). In these studies, sterile protection is defined as the absence of infected red blood cells on thick blood smears during the 21-day follow-up after challenge. In the first study (ClinicalTrials.gov registration number NCT00442377), 10 healthy malaria-naive Dutch volunteers received 15 infective mosquito bites and were completely protected (13, 22); plasma samples from 9 of these volunteers were available for this study. In the second study (ClinicalTrials.gov registration number NCT01236612), 5 of the CPS-immunized volunteers were fully protected. However, for the purpose of this study, one immunized individual later developed parasitemia on day 21 after sporozoite challenge (retrospectively detected by quantitative PCR [qPCR]) and therefore was considered nonprotected (18). In the third study (ClinicalTrials.gov registration number NCT01218893), healthy volunteers received CPS immunization with bites from graded numbers of P. falciparum-infected mosquitoes (three rounds of infection by 15 mosquito bites [3 × 15] [*n* = 5], 3 × 10 [*n* = 9], or 3 × 5 [*n* = 10]); 4 out of 5 subjects in the 3 × 15 group, 8 out of 9 subjects in the 3 × 10 group, and 5 out of 10 subjects in the 3 × 5 group were protected against challenge infection (16). Citrate plasma samples from all three studies were collected longitudinally at three time points (7 days before CPS immunization [I1-7], 1 day before CHMI challenge [C-1], and 35 days postchallenge [C+35]), probed, and analyzed for this study. In total, we had 30 protected and 8 nonprotected individuals from these studies.

### Microarray construction.

We constructed whole Plasmodium falciparum 3D7 (*Pf*3D7) proteome microarrays with 7,455 full-length or segmented protein features displaying about 91% of the P. falciparum proteome (Antigen Discovery, Inc., Irvine, CA). Sequence-verified polypeptides were printed as *in vitro* transcription and translation (IVTT) reactions as previously described (39). These 7,455 P. falciparum open reading frames (ORFs) represent 4,768 unique genes. T7 promoter plasmids encoding each protein used in the IVTT reactions were obtained using a high-throughput cloning method described previously (18). Quality control of array slides was performed as described previously (22), with a >97% protein expression efficiency of the IVTT reactions spotted.

### Protein microarray probing.

P. falciparum-specific plasma antibodies induced by CPS immunization bind to their cognate spotted IVTT antigen/polypeptide, which is detected and quantified using fluorescent secondary antibodies, akin to Western blotting. Plasma samples were diluted 1:200 in protein array blocking buffer (Whatman, Inc., Sanford, ME) supplemented with a 10% (vol/vol) E. coli DH5α lysate (MCLAB, San Francisco, CA) and incubated on arrays overnight at 4°C. Blocking of anti-E. coli antibodies prevents binding of any anti-E. coli antibodies in plasma samples and thus reduces the background. Slides were blocked in protein array blocking buffer (Whatman, Inc., Sanford, ME) for 30 min and then incubated with plasma for 2 h at room temperature. Bound antibodies were detected by incubation with biotin-SP-conjugated affinity-purified goat anti-human IgG Fc fragment-specific secondary antibody (Jackson ImmunoResearch, West Grove, PA) diluted 1:200 in protein array blocking buffer (Whatman, Inc., Sanford, ME), followed by streptavidin-conjugated SureLight P-3 (Columbia Biosciences, Columbia, MD). The microarrays were scanned with a GenePix 4200 AL microarray scanner (Molecular Devices, Sunnyvale, CA). Microarray spot intensities were quantified using the ProScanArray Express microarray analysis system (PerkinElmer LAS, Waltham, MA) with spot-specific background correction. The raw values from array scans were the mean intensities of all the pixels in printed spots after subtracting surrounding background intensities for each protein or negative control. IVTT controls, or ‘‘no-DNA’’ controls, are negative-control spots consisting of IVTT reaction mixtures without the addition of a plasmid template. The IVTT control spots on each array were averaged, and this value was subtracted from the values for every other spot on the array. Samples probed on different days and from different batches of slides were correlated, showing that slide printing and probing were reproducible.

### Data normalization, analysis, and statistics.

All analyses were performed using the R statistical environment and “ggplot2,” an R package used to visualize the data (62, 63). Univariate logistic regression was used to examine the association of protection status and reactivity against malaria antigens. To account for differences in baseline antibody responses seen among the different immunized individuals and to detect only antibodies induced by CPS immunization, preimmunization (I1-7), baseline antibody levels for each antigen were subtracted from the data generated using matched subsequent prechallenge (C-1) and postchallenge (C+35) plasma samples for each individual. The analysis reported here uses a new metric for defining reactive antigens. We used a computational approach using the R package “mixtools” (64) to quantify each volunteer’s antibody responses at given time point. This package allows for the clustering of observations (antibody responses) that are assumed to have arisen from a mixture of finite distributions (positive and negative antibody responses).

In previous studies from our group, we used the average of negative-control spots (IVTT control spots [no-DNA negative controls]) from all volunteers to create a global median cutoff point; antibody responses above this cutoff were classified as seropositive (positive antibody response), and those below it were classified as seronegative (negative antibody response) (19–21). In present study, the mixture model was constructed using each subject’s antibody responses at all the time points (seroreactivity) against all antigens (7,455 antigens). The two-component mixture model identifies the positive and negative antibody distributions, thereby allowing us to set the cutoff point as the mean of the negative antibody signal distribution plus 3 standard deviations (SD) (see Fig. S1 in the supplemental material). For better visualization of the low-frequency high-signal-intensity data points, the horizontal axis scale was square-root transformed using the function sqrt_trans() in the “scales” R package. Using this cutoff point, we identified seropositive and seronegative antibody responses (responses above or below the individuals’ cutoff, respectively). Importantly, this tool takes the intrinsic interindividual baseline variability in each subject’s specimen into consideration, and therefore, the unbiased cutoff point varies between individuals. Immunogenic antigens were defined as antigens with a seropositive antibody response and a 2-fold increase in the antibody response at C-1 compared to that at I1-7. For each subject, the total numbers of antigen targets that were immunogenic (breadth) were calculated. For protein features to be considered reactive at the population level, they had to be immunogenic in more than 15% of all the immunized subjects (Table S1). Only antigens considered reactive based on these criteria were used for subsequent analysis.

The reactive antigens were tested for differences in antibody levels and breadth counts comparing CPS-immunized protected and nonprotected individuals using a two-tailed Wilcoxon rank sum test. A Kruskal-Wallis rank sum test was used to test for differences in variables that had more than two groups. Results were considered statistically significant when *P* values were <0.05. *P* values were corrected for multiple testing by adjusting the false discovery rate (FDR) using the Benjamini-Hochberg procedure as applied by the p.adjust() R function (65). Furthermore, we tested each individual antibody’s performance in predicting protection by constructing receiver operating characteristic (ROC) curves and calculating the area under the curve (AUC). Protected individuals were labeled with positive class labels; therefore, an AUC value of >0.5 showed that the specific antibody levels were associated with protection and were higher in protected than in nonprotected volunteers. An AUC value of <0.5 indicated that the specific antibody levels were lower in protected volunteers and therefore associated with susceptibility to malaria. To test for generalizability of our regression model, we also performed leave-one-out cross-validation (LOOCV). LOOCV was done for each protective/susceptible antigen; one sample was omitted from the data set (test set), and a regression model was built on the remaining data (training set). The model is tested for generalizability by predicting the status of the omitted sample. This is repeated, each time with a different sample left out, until all the samples have been omitted once.

In order to assess whether the protective seropositivity counts (numbers of seropositive responses to antigens associated with protection) were more important for protection than susceptible seropositivity counts, we determined the difference between protective seropositivity counts and susceptible seropositivity counts and then used this difference to construct an ROC curve. LOOCV analysis was also performed for this model. Comprehensive descriptions of the cohorts compared are provided in the corresponding figure legends.

### Plasmodium falciparum protein expression.

There are 5,305 P. falciparum 3D7 protein-coding genes identified in PlasmoDB (6) (version 38; http://plasmodb.org/plasmo/). All gene annotations with tandem mass spectrometry (MS/MS) evidence of expression were downloaded from PlasmoDB. MS/MS studies have identified 248,678 peptides that map to 4,216 P. falciparum proteins. Due to the fact that some of the P. falciparum stages are not easily accessible, MS/MS studies have mostly been done on the following parasite preparations: schizonts, trophozoites, merozoites, infected erythrocytes, or sporozoites. For ease of analysis, all proteins identified in schizont, trophozoite, merozoite, and infected erythrocyte samples were simply denoted blood-stage proteins, while all proteins identified in sporozoite parasite sample preparations were denoted sporozoites. To get an overall idea of the stage of expression for the proteins recognized by CPS-immunized subjects, we compared the 4,216 proteins with MS/MS evidence with unique proteins printed on the array and recognized by either at least 25% of the responder groups or at least 25% of protected and nonprotected subjects.

10.1128/mSphereDirect.00027-19.6 FIG S3 Reactivity against representative antigens of early- and mid/late-liver*-*stage development of parasites. Antibody reactivity in low responders (light gray) (*n* = 16) was compared to that in medium/high responders (dark gray) (*n* = 25) by a Wilcoxon rank sum test. Dots show individual data points, center lines show the medians, box limits indicate the 25th and 75th percentiles, and whiskers extend 1.5 times the IQR. Download FIG S3, TIF file, 0.3 MB. Copyright © 2019 Obiero et al. 2019 Obiero et al. This content is distributed under the terms of the Creative Commons Attribution 4.0 International license.
