# Supplementary figures and images for: Antibody Biomarkers Associated with Sterile Protection Induced by Controlled Human Malaria Infection under Chloroquine Prophylaxis
Source: mSphere. 2019 Feb 20;4(1):e00027-19. doi: 10.1128/mSphereDirect.00027-19 (PMC6382972; doi:10.1128/mSphereDirect.00027-19)

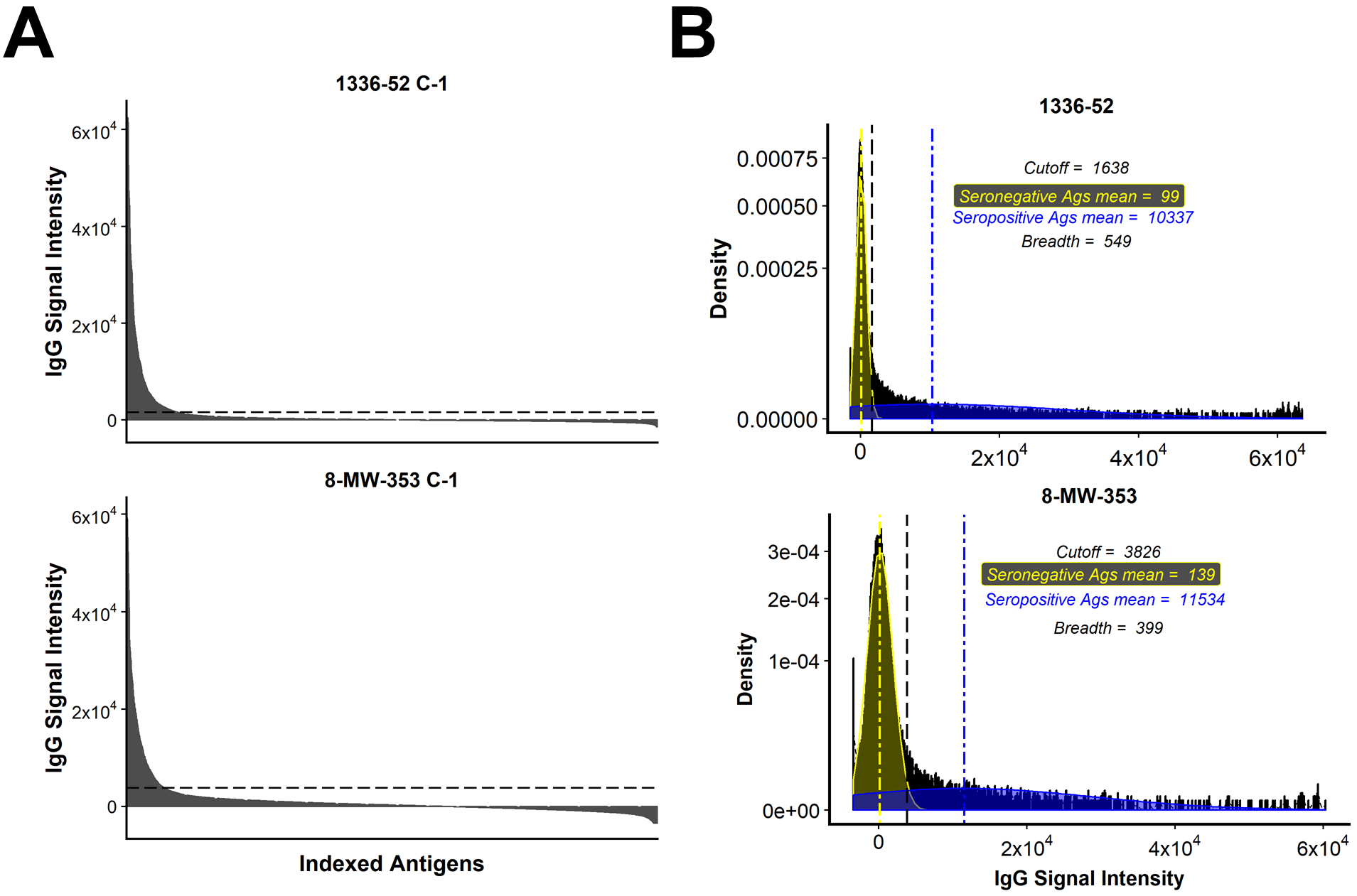

Supplement: FIG S1 [file mSphereDirect.00027-19-sf001.tif]

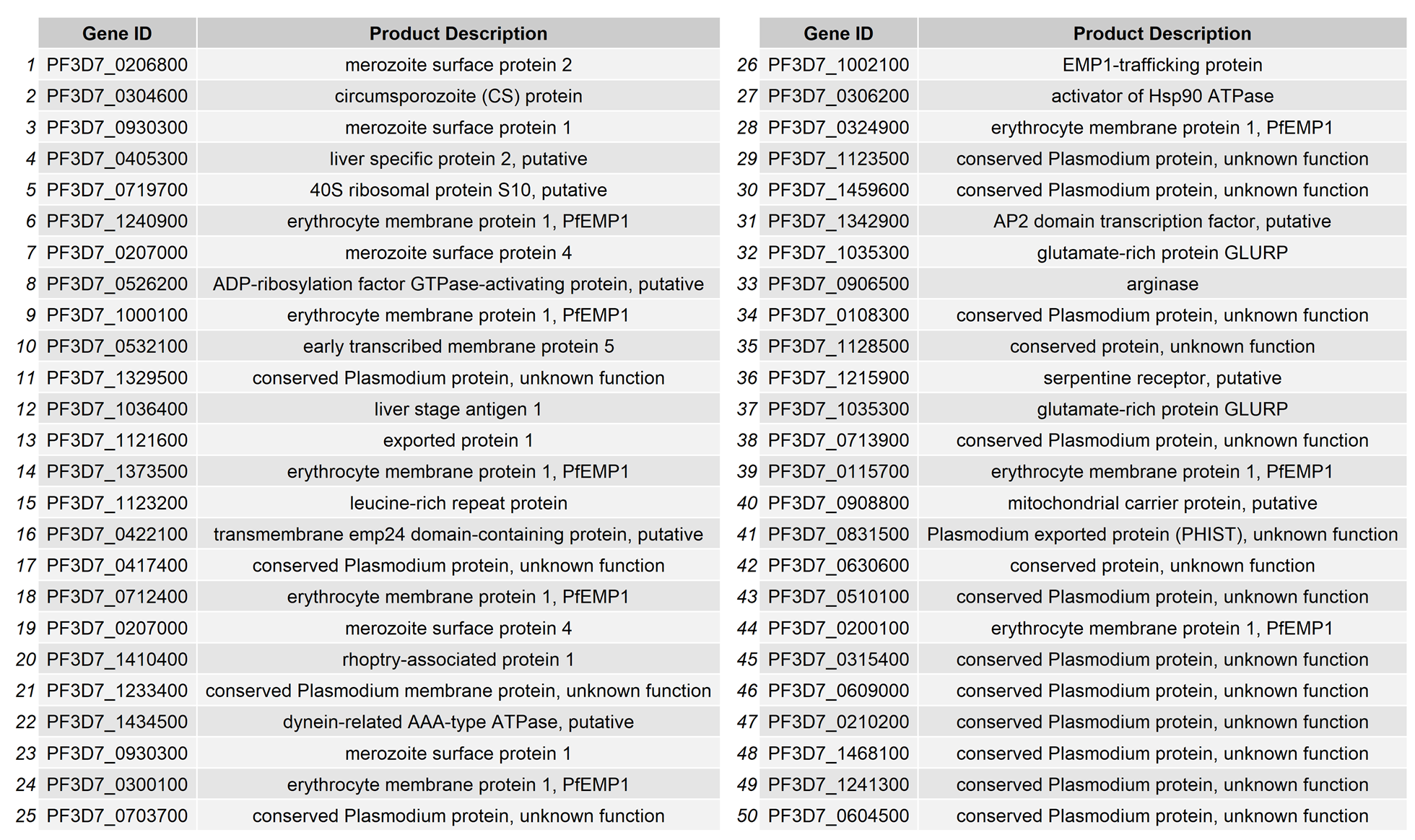

Supplement: TABLE S2 [file mSphereDirect.00027-19-st002.tif]

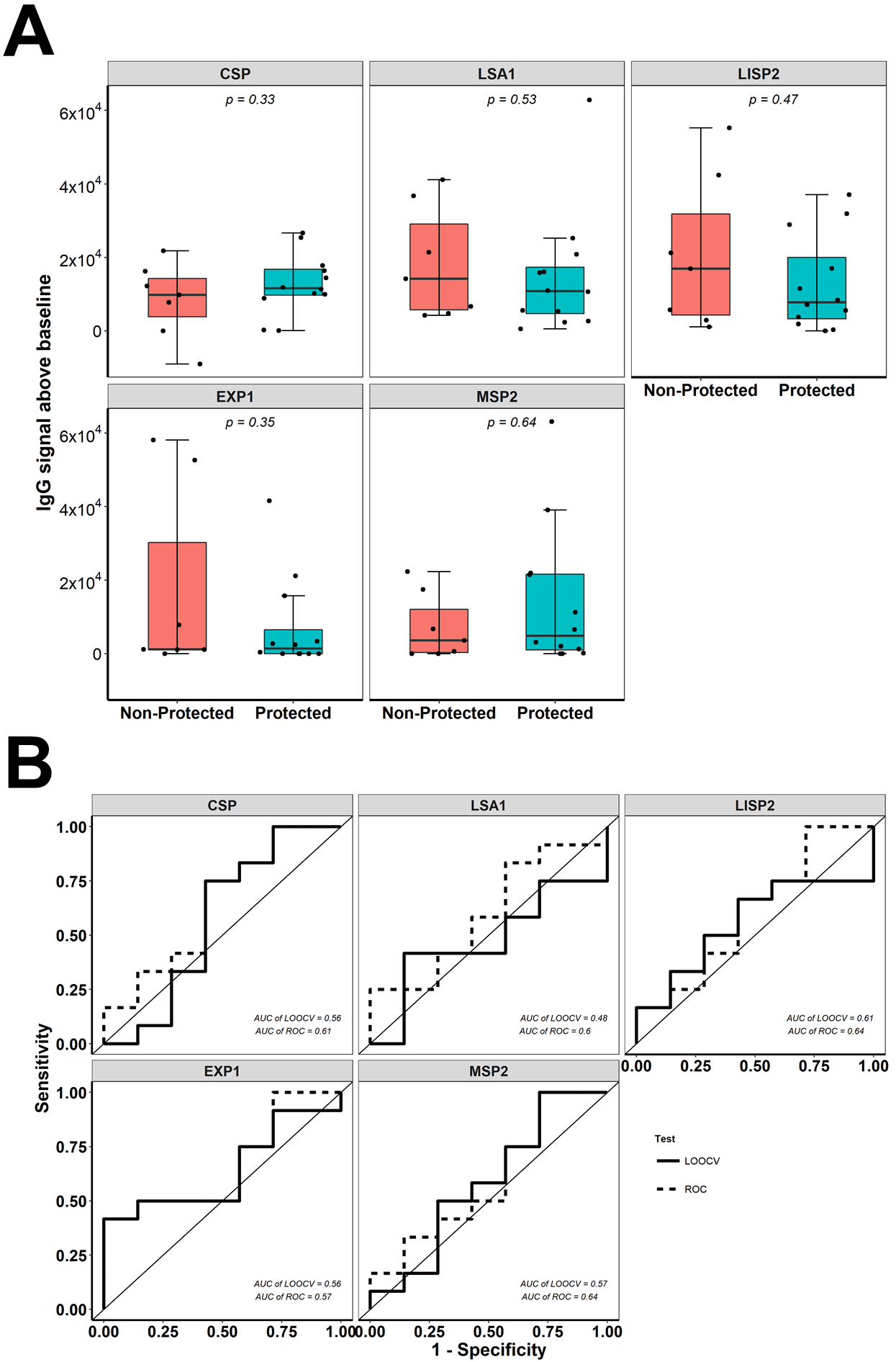

Supplement: FIG S2 [file mSphereDirect.00027-19-sf002.tif]

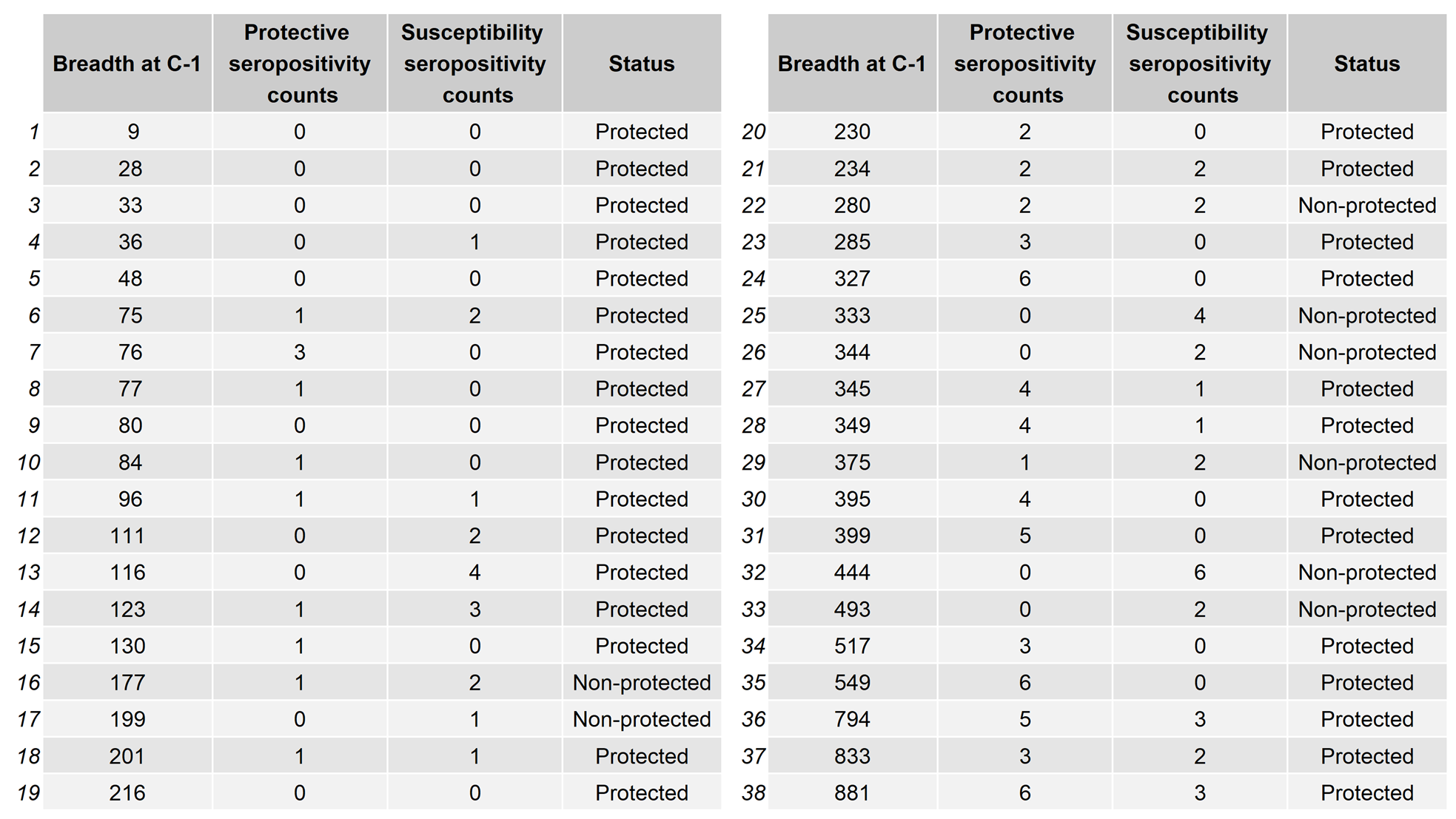

Supplement: TABLE S3 [file mSphereDirect.00027-19-st003.tif]

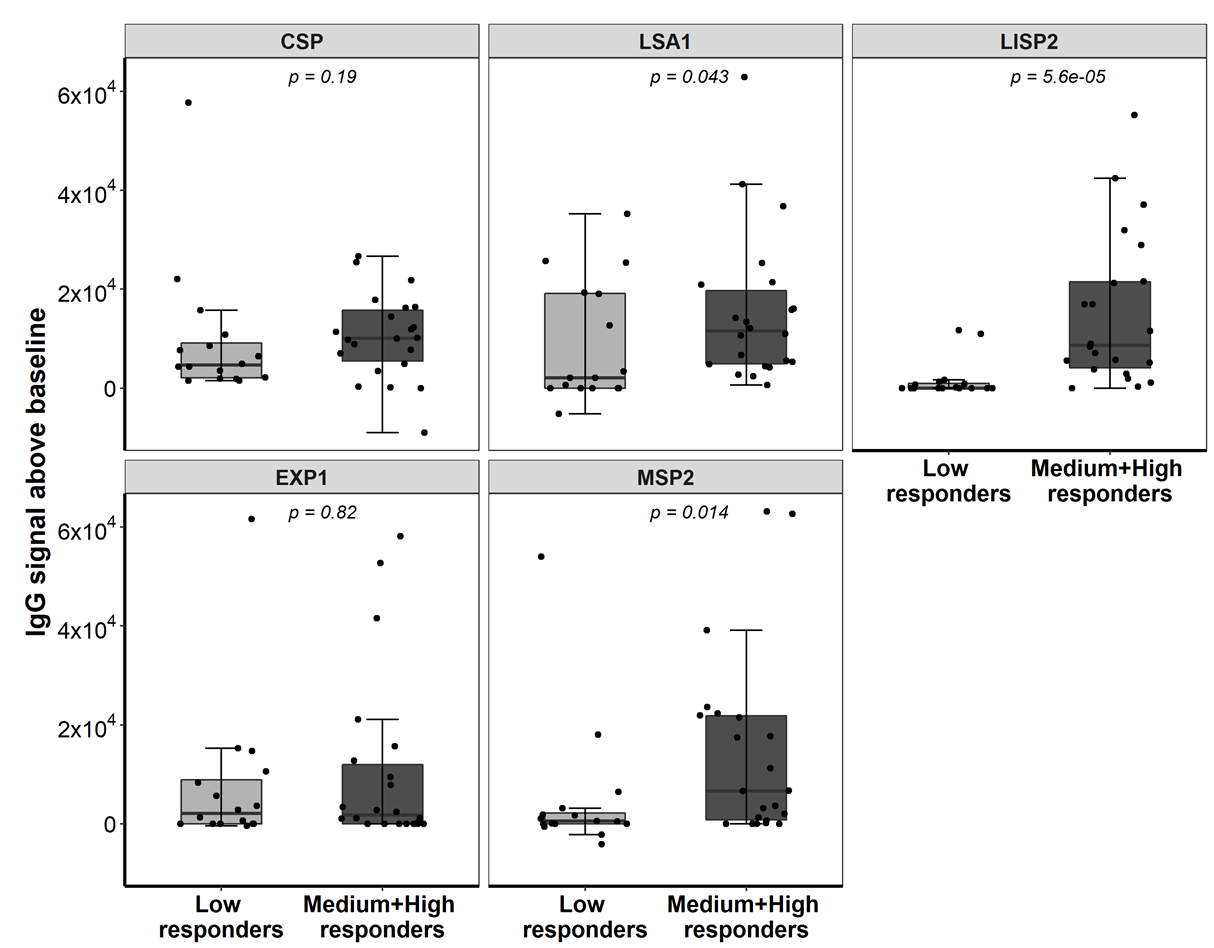

Supplement: FIG S3 [file mSphereDirect.00027-19-sf003.tif]
